# Virtual Screening of Benzimidazole Derivatives as Potential Triose Phosphate Isomerase Inhibitors with Biological Activity against *Leishmania mexicana*

**DOI:** 10.3390/ph16030390

**Published:** 2023-03-03

**Authors:** Lenci K. Vázquez-Jiménez, Alfredo Juárez-Saldivar, Manuel J. Chan-Bacab, Timoteo Delgado-Maldonado, Luis D. González-Morales, Isidro Palos, Eyra Ortiz-Pérez, Edgar E. Lara-Ramírez, Esther Ramírez-Moreno, Gildardo Rivera

**Affiliations:** 1 Laboratorio de Biotecnología Farmacéutica, Centro de Biotecnología Genómica, Instituto Politécnico Nacional, Reynosa 88710, Mexico; 2 Centro de Investigación en Microbiología Ambiental y Biotecnología, Universidad Autónoma de Campeche, Campeche 24039, Mexico; 3 Unidad Académica Multidisciplinaria Reynosa-Rodhe, Universidad Autónoma de Tamaulipas, Reynosa 88779, Mexico; 4 Escuela Nacional de Medicina y Homeopatía, Instituto Politécnico Nacional, Ciudad de Mexico 07320, Mexico

**Keywords:** virtual screening, molecular docking, benzimidazole, triosephosphate isomerase, *Leishmania mexicana*

## Abstract

*Leishmania mexicana* (*L. mexicana*) is a causal agent of cutaneous leishmaniasis (CL), a “*Neglected disease*”, for which the search for new drugs is a priority. Benzimidazole is a scaffold used to develop antiparasitic drugs; therefore, it is interesting molecule against *L. mexicana*. In this work, a ligand-based virtual screening (LBVS) of the ZINC15 database was performed. Subsequently, molecular docking was used to predict the compounds with potential binding at the dimer interface of triosephosphate isomerase (TIM) of *L. mexicana* (*Lm*TIM). Compounds were selected on binding patterns, cost, and commercial availability for in vitro assays against *L. mexicana* blood promastigotes. The compounds were analyzed by molecular dynamics simulation on *Lm*TIM and its homologous human TIM. Finally, the physicochemical and pharmacokinetic properties were determined *in silico*. A total of 175 molecules with docking scores between −10.8 and −9.0 Kcal/mol were obtained. Compound **E2** showed the best leishmanicidal activity (IC_50_ = 4.04 µM) with a value similar to the reference drug pentamidine (IC_50_ = 2.23 µM). Molecular dynamics analysis predicted low affinity for human TIM. Furthermore, the pharmacokinetic and toxicological properties of the compounds were suitable for developing new leishmanicidal agents.

## 1. Introduction

Cutaneous leishmaniasis (CL) is a disease characterized by solitary or multiple ulcerated skin lesions [1]. The causative agents are Leishmania parasites transmitted by phlebotomid sandflies. In America, *Leishmania mexicana* (*L. mexicana*) and other subgenera are predominant agents [2,3]. Although CL is not a fatal disease, it does cause disability and permanent scars [4]. The World Health Organization (WHO) recommends pentavalent antimonials as first-line drug treatment [5]. Alternatives include the use of liposomal amphotericin B. However, these compounds are toxic, have low efficacy in the chronic phase of the disease, and cannot be widely used due to their high cost and administration requirements [6]. These factors potentiate the search for new and more effective drugs against CL.

In the last decades, different targets have been considered to develop new leishmanicidal agents, such as trypanothione reductase [7], cysteine proteinases [8], glyceraldehyde-3-phosphate dehydrogenase [9], and triosephosphate isomerase (TIM) [10]. The latter is involved in the fifth step of the glycolysis pathway and is essential in energy production [11,12]. TIM has structural differences with its human homologue, making the obtention of selective inhibitors possible. It has been widely studied in various protozoa as a pharmacological target [13,14,15,16], and, in this sense, the TIM of *L. mexicana* (*Lm*TIM) can be considered a drug target for developing new leishmanicidal agents.

Benzimidazole is an aromatic heterocyclic compound with a wide range of biological activities [17] as an antiviral [18], an anthelmintic [19], an antimicrobial [20], an antiparasitic [21], and others [22,23]. Hybrids of benzimidazole and pentamidine derivatives have been tested against *L. mexicana* [24]. For example, Torres-Gómez et al., showed that the compounds **C1** and **C2** (Table 1) were more active (IC_50_ = 0.712 and 0.368 μM, respectively) than pentamidine (IC_50_ = 9.568 μM). Subsequently, Nieto-Meneses et al. [25] obtained compounds **C3** and **C4** (Table 1) with better leishmanicidal activity (IC_50_ = 2.62 and 3.21 μM, respectively) than miltefosine (IC_50_ = 15.34 μM) and lower cytotoxicity (SI = 91.76 and 317.75, respectively) than miltefosine and amphotericin B (SI = 10.23 and 6.5, respectively). These studies showed the effectiveness of benzimidazole-containing compounds as leishmanicidal agents.

In this work using the benzimidazole scaffold, a ligand-based virtual screening (LBVS) was performed of the ZINC15 database. Then, a molecular docking analysis was performed to identify potential binding at the interface of *Lm*TIM. The selected compounds were further evaluated against the promastigote form of *L. mexicana*. In addition, molecular dynamics simulations were carried out to predict the stability of the compounds evaluated in complex with *Lm*TIM and their affinity for human TIM (*Hs*TIM). Finally, the pharmacokinetic and physicochemical properties were predicted.

## 2. Results and Discussion

### 2.1. Binding Site Prediction and Molecular Docking of Control Ligands

Six compounds with leishmanicidal and/or inhibitory activity against *Lm*TIM were selected as control compounds [24,25,26] and analyzed by molecular docking at the interface of the *Lm*TIM protein. Compounds **C1**–**C4** are benzimidazole derivatives, **C5** is a benzothiazole derivative, and **C6** is a quinoline derivative. Compound **C2** has the best leishmanicidal activity (IC_50_ = 0.36 μM), and **C5** shows a 70% inhibition on the *Lm*TIM (Table 1) [10].

The binding site of the control compounds on the *Lm*TIM protein (ID: 1AMK) for molecular docking analysis was established with the DoGSiteScorer tool. This pocket detection and analysis tool identifies potential binding pockets in protein structures. By default, it provides a simple drugability score for each pocket, based on a linear combination of three descriptors (volume, hydrophobicity, and enclosure) [27]. The results show the dimer interface as the best binding site with a drug score greater than 0.5.

**Figure 1:**
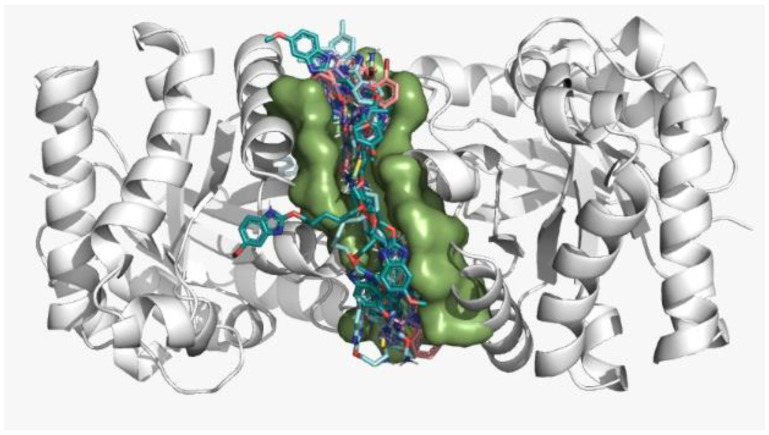
Prediction of the binding site of control compounds by the DoGSiteScorer web server and blind molecular docking on *Lm*TIM. The protein is represented in gray color, and the dimer interface is represented in green color.

Additionally, a blind molecular docking was performed to assess the potential binding of each control compound at the site predicted by the DoGSiteScorer tool (assigning a docking score) [28]. In this study, blind molecular docking showed docking scores of −9.2 to −7.1 Kcal/mol of the control compounds at the *Lm*TIM dimer interface (Table 1). Compound **C6** had the lowest docking score of −9.2 Kcal/mol. The other five control compounds showed docking scores from −7.8 to −7.1 Kcal/mol (Figure 1). The interface, contrary to the active site, is a non-conserved site. It has been studied because it causes a selective allosteric enzymatic inhibition in protozoa by promoting the destabilization of the protein*’*s quaternary structure, leading to death [29,30].

The interaction profile of the six control compounds (Table 1) shows hydrophobic interactions, hydrogen bonding, and π-stacking interactions. Hydrophobic interactions with the Ile69, Glu105, and Ile109 residues of the B monomer predominated in the six control compounds, except Ile69 with the compound **C5**. These interactions have been reported to play a key role in inhibitory activity [16]. Additionally, the compounds **C1**, **C2,** and **C3** present hydrogen bonds with Lys113 and Gln112 in monomer B, among others; compounds **C4** and **C5** were hydrogen-bonded only with Tyr103 in monomer A. The compound **C6** with the best binding energy showed no hydrogen-bonding or π-stacking interactions. Only compounds **C1** through **C4** exhibited hydrogen-bonding interactions and π-stacking with at least some of the amino acid residues, Tyr102 in monomer A, and Phe75B or Tyr103 in monomer B. Interestingly, the interactions with amino acid residues at the TIM interface of other protozoa, such as Tyr102, Tyr103, Ile69, Asn67, Phe75, Thr70, Glu105, and Lys113, were also observed in other studies [31,32]. These results support the dimer interface as the best binding site for further virtual screenings. Additionally, in vitro enzymatic studies report that benzimidazole derivatives have up to 69% inhibition percentages on the TIM enzyme [30,33], supporting continuing efforts to search for this type of inhibitor.

### 2.2. LBSV in ZINC15

The LBVS in the ZINC15 database (750 million compounds) obtained 67,141 compounds by a substructure search using the benzimidazole scaffold. Applying Lipinski*’*s rule as inclusion criteria, 53,410 compounds were filtered and selected for the molecular docking analysis. The results showed that 175 compounds had a docking score between −10.8 and −9.0 Kcal/mol, which was near and higher than the docking score of the control compound **C6** ligand (−9.2 Kcal/mol). The compounds were grouped using two criteria: (a) based on the protein-ligand interaction profile (PLIP) and the scikit-learn library, and (b) on structure similarity descriptors using the algorithms of DataWarrior software. Table 2 shows the lead compounds of each group, arranged by their interaction profile, the number of compounds obtained, and the docking score. Group two had the highest number of compounds (*n =* 24), while group eight had the lowest number of compounds (*n =* 9) (full groups are shown in Supplementary Material S1).

In protozoa, the interface site is hydrophobic [33]; on the other hand, benzimidazole is a nitrogenous heterocycle with two equivalent tautomeric forms, in which the hydrogen atom can be located in any of the two nitrogen atoms, facilitating the formation of hydrogen bonds [34]. This block can also act as a proton acceptor or donor, binding to the protein through various interactions such as hydrogen bonds, van der Waals forces, and π–π stacking, among others [35]. In this sense, the lead compounds showed hydrophobic interactions, hydrogen bonding, and π-stacking. The hydrophobic interactions occurred with the amino acids Tyr102 or Tyr103 in the A monomer and Ile69 and Phe75 in the B monomer in most of the compounds. Hydrogen bonding interactions with the amino acids Gln112, Lys113, and Glu105 in the B monomer were the most common in the compounds. π-stacking interactions were also present with the amino acids Tyr102 and Tyr103 in monomer A and Phe75 in monomer B. Compound **P4** with the lowest docking score (−10.3 Kcal/mol) did not show π-stacking interactions; however, it showed four additional hydrophobic interactions (Tyr103 in monomer A, Ile109, Gln112 and Glu116 monomer B) and two hydrogen bond interactions with the amino acids Ala70 and Arg99 in monomer B, which have been described as promoting the formation of more stable ligand-protein complexes [10]. In addition, they coincide with interactions presented by the control compounds (Table 1).

Table 3 shows the 10 groups obtained by structure, the number of compounds in each group, and the lead compound with the best docking score (−10.8 and −9.0 Kcal/mol). This grouping was based on the type of structure and how similar they were to each other. Group three presented the largest number of compounds (*n =* 102), while the rest presented 1 and up to 26 compounds per group (the entire groups are shown in Supplementary Material S1).

The interactions that predominated were hydrophobic with the amino acid residues Ile69, Ile109, and Phe75 in the B monomer in five compounds (**E1**, **E2**, **E6**, **E7**, and **E8**). The hydrogen bonding interaction with the Gln112 amino acid residue in the B monomer was the most common in most compounds and presented π-stacking interactions with the amino acid residues Tyr102 and Tyr103 in the A monomer and Phe75 in the B monomer. Only compound **E9** presented a salt-bridge type interaction with the Lys113 amino acid residue in monomer B. In contrast, compound **E10** showed an interaction of the halogen with the amino acid residue Lys71 in monomer B. Considering the described binding patterns, and the commercial availability and price, compounds **P9** (Table 2) and **E2** (Table 3) were purchased to determine their leishmanicidal activity.

### 2.3. Leishmanicidal Activity

The half-maximal inhibitory concentration (IC_50_) of compounds **P9** and **E2** against *L. mexicana* promastigotes is shown in Table 4. Compound **E2** had better leishmanicidal activity against the promastigotes form of *L. mexicana* (IC_50_ *=* 4.04 µM) than **P9** (IC_50_ > 148.63), and similar to the reference drug pentamidine (IC_50_ *=* 2.23 µM).

### 2.4. Molecular Dynamics Analysis

Molecular dynamics simulation was performed to predict the stability of the ligand–protein complex due to the leishmanicidal effect of compounds **P9** and **E2**, their high binding affinity, and an interaction profile similar to the control compounds at the *Lm*TIM interface. The apo form protein (*Lm*TIM) and the control compound **C6** in complex with *Lm*TIM were also analyzed, considering previous studies [36,37].

**Figure 2:**
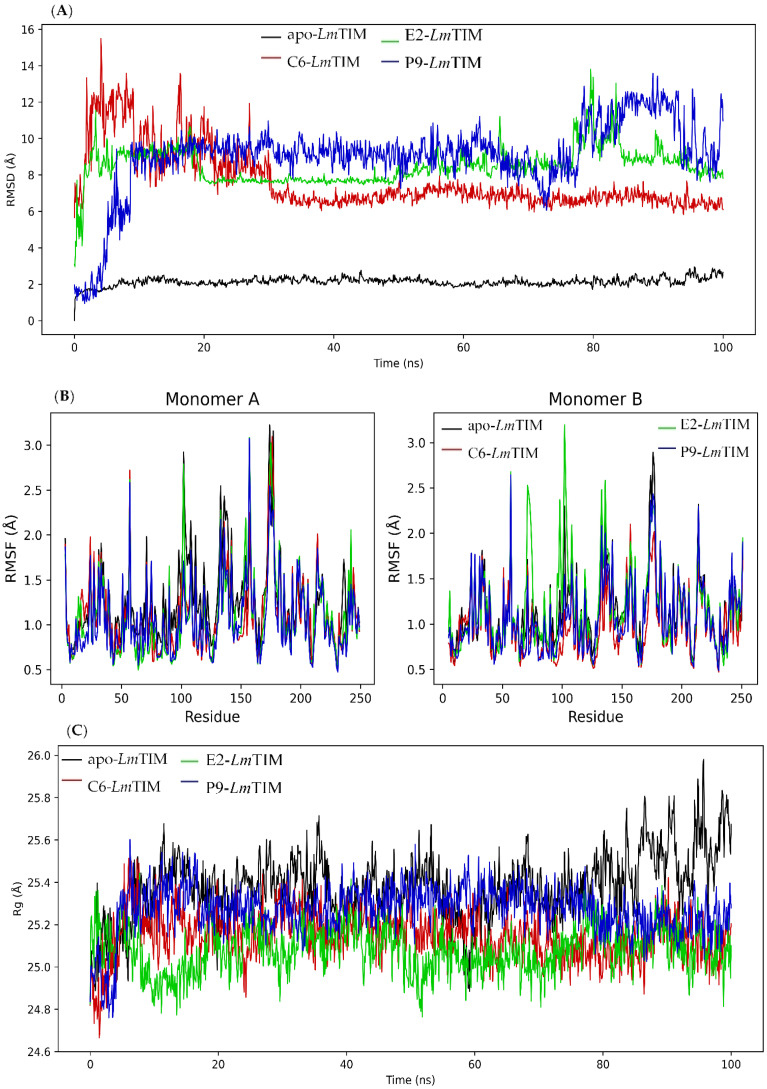
Molecular dynamics simulation of benzimidazole derivatives (**E2** and **P9**) and control compound **C6** in complex with *Lm*TIM. (**A**) Plot of RMSD of the three complexes and apo-*Lm*TIM. (**B**) Diagram of the RMSF of the three complexes and apo-*Lm*TIM. (**C**) Diagram of Rg of the three complexes and apo-*Lm*TIM.

The analysis of the apo-*Lm*TIM protein showed a RMSD with a minimum of 0.01 Å and a maximum of 3.12 Å, as well as a mean oscillation of 2.13 Å. The **C6**-*Lm*TIM complex had an RMSD of 5.62 to 15.77 Å and a mean oscillation of 7.62 Å. The **E2**-*Lm*TIM complex fluctuated from 2.61 Å to 13.86 Å with a mean oscillation of 8.48 Å. The **E2**-*Lm*TIM complex showed a change in the initial position in the first 20 ns (Figure S1 of the Supplementary Material). After that, it remained stable until approximately 80 ns (with a RMSD mean of 3.65 Å), again observing a fluctuation between 80-82 ns and continuing stable until 100 ns. On the other hand, the **P9**-*Lm*TIM complex showed a fluctuation of 0.92 to 14.25 Å and a mean oscillation of 9.01 Å (Figure 2A). The RMSD in the first 10 ns had a large fluctuation that can be attributed to a change in the initial position of the compound at the binding site (Figure S2 of the Supplementary Material). Subsequently, the complex had a stability of 10 to 80 ns (with a RMSD mean of 1.51 Å).

In general, the compounds **P9**, **E2**, and the control ligand in complex with *Lm*TIM had a fluctuating behavior that suggests a change in the initial binding position [38,39]. The complex with the lowest RMSD and minimal differences between oscillations has been described as the most stable [40]. In this sense, the **E2**-*Lm*TIM complex showed less oscillation and better stability after 20 ns than the **P9**-*Lm*TIM complex. Both complexes showed a greater oscillation than the **C6**-*Lm*TIM complex. Although an interaction profile analysis of molecular dynamics allowed us to observe that some initial interactions were maintained in both complexes (Figures S3 and S4 of the Supplementary Material).

The RMSF was also analyzed during molecular dynamics simulation (Figure 2B). The RMSF is a measure of the variation in the structure of a protein over time, calculated from a trajectory generated by molecular dynamics simulation. This measure allows evaluation of the stability and flexibility of the protein, as well as the influence of the interaction between the protein and the ligand on the stability of the system [41,42]. The RMSF results showed a similar fluctuation pattern between the apo-*Lm*TIM and the protein in complex with the ligands. The apo-*Lm*TIM protein showed a RMSF with a minimum of 0.52 Å, a maximum of 3.23 Å, and a mean oscillation of 1.20 Å (Figure 2B). The **C6**-*Lm*TIM complex had a RMSF of 0.47 to 3.10 Å with a mean oscillation of 1.05 Å. The **E2**-*Lm*TIM complex showed a fluctuation from 0.50 Å to 3.19 Å with a mean oscillation of 1.14 Å. The **P9**-*Lm*TIM complex fluctuated from 0.47 to 3.08 Å with a mean oscillation of 1.07 Å.

We observed, in the RMSF calculation, that the high fluctuation in some regions according to the RMSD pattern, may be due to residues that are in constant movement in both monomers of the protein, such as the loops, with this movement being a factor that contributes to the elevation of the RMSD values. This finding has been described in the TIM of other species, such as *Trypanosoma cruzi* with approximately 82% identity to the *Lm*TIM interface [43,44].

Finally, the rotation radius (Rg) of apo-*Lm*TIM and the protein in complex with the compounds (**C6**, **E2**, and **P9**) (Figure 2C) was analyzed to predict the structural variations of the protein during the molecular dynamics analysis [45]. The free folding of *Lm*TIM maintains an almost constant fluctuation between 24.79 and 25.98 Å with a difference in the oscillation of 1.19 Å during the 100 ns analyzed with a mean of 25.38 Å (Figure 2C). The **C6**-*Lm*TIM complex fluctuated between 24.62 and 25.64 Å with a difference of 1.02 Å. The **E2**-*Lm*TIM complex fluctuated from 24.62 to 25.45 Å with a difference of 0.83 Å, and the **P9**-*Lm*TIM complex from 24.71 to 25.67 Å with a difference of 0.95 Å. Similar Rg values in the three complexes and greater compactness in their dynamics suggest that ligand interactions do not influence the variation of the *Lm*TIM structure [46].

### 2.5. ADMET In Silico

Finally, an in silico analysis of the molecular, pharmacokinetic (SwissADME), and hepatotoxicity (ProTox-II) physicochemical properties of compounds **P9** and **E2** was performed. The results are shown in Table 5.

Compounds **P9** and **E2** complied with the Lipinski’s rule, which plays an important role in drug discovery and development [47,48]. On the other hand, the predictive study showed high human intestinal absorption of the two compounds. Furthermore, moderate solubility was predicted. However, these compounds are substrates of P-glycoprotein, an efflux pump that plays an important role in normal physiological detoxification and is associated with drug resistance [49]. Some antibiotics and anticancer drugs have been described that can be P-glycoprotein substrates and be used [50].

Hepatotoxicity predictions were negative, and regarding CYP450 inhibition, they showed that, according to structure, compound **E2** inhibits two isoforms (1A2 and 2C19). Compound **P9** is likely an inhibitor of the four CYP450 isoforms analyzed, giving rise to drug–drug interactions. It would be important to search for optimization in the bioavailability of this class of compounds with leishmanicidal potential.

### 2.6. Molecular Docking on HsTIM

**Figure 3:**
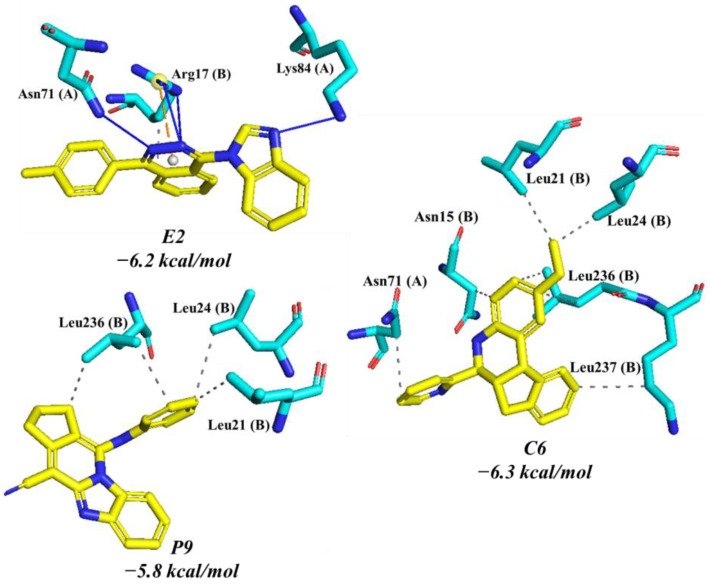
Interaction profile and docking score of compounds **C6**, **E2** and **P9** at the *Hs*TIM interface. Hydrogen bonds are shown as blue lines, hydrophobic interaction as gray dashed lines, and salt bridge interactions as yellow dashed lines. Interactions were obtained using the PLIP online server.

Compounds **C6**, **E2**, and **P9** were evaluated against *Hs*TIM by molecular docking (Figure 3) to establish the potential selectivity. Control compound **C6** showed a docking score of −6.3 Kcal/mol and seven hydrophobic contacts with Asn71 in monomer A and Asn15, Leu21, Leu24, Leu236, Leu237 in monomer B. Compound **P9** exhibited a docking score of −5.8 Kcal/mol. The phenyl ring of **P9** was oriented towards the leucine triad (Leu21, Leu24, and Leu236 in the B monomer), which led to the formation of hydrophobic interactions between the carbons of the leucine residues and the carbons of the aromatic ring. Compound **E2** presented a docking score of −6.2 Kcal/mol. It only showed interactions with three residues forming four hydrogen bonds, a hydrophobic interaction, and a salt bridge with Arg17, Asn71, and Lys84 in monomer A (Figure 3).

### 2.7. Molecular Dynamics Simulation on HsTIM

**Figure 4:**
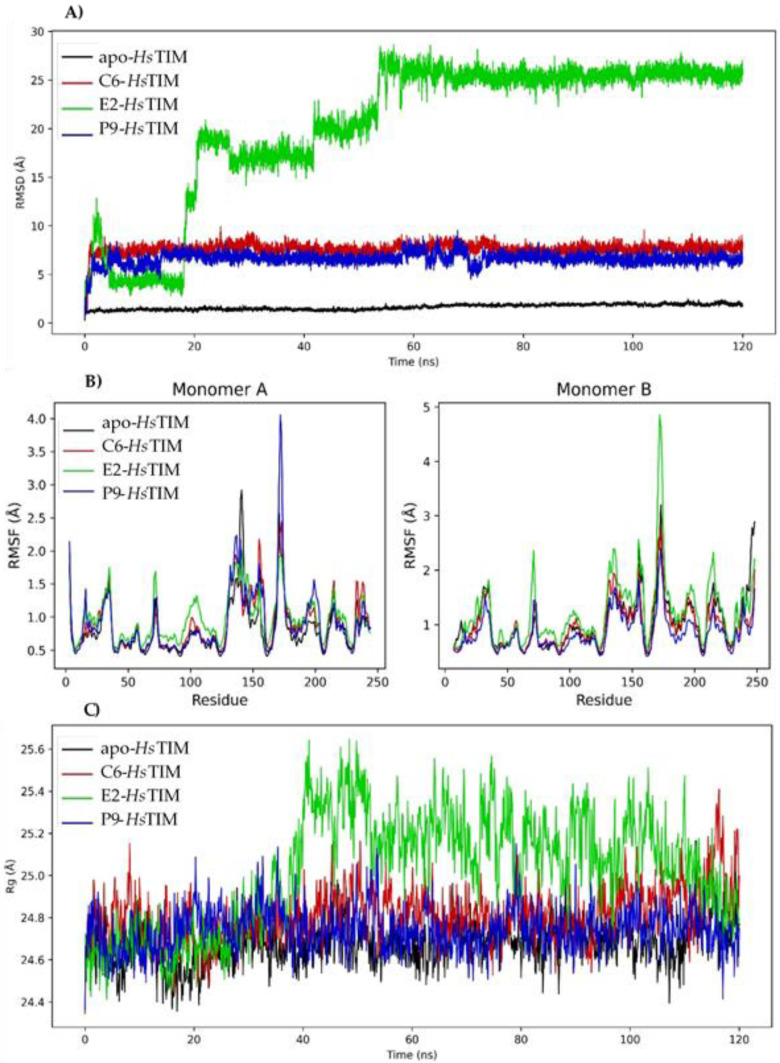
Molecular dynamics simulation of benzimidazole derivatives in complex with *Hs*TIM. (**A**) Plot of RMSD of the three complexes and apo-*Hs*TIM. (**B**) Diagram of the RMSF of the three complexes and apo-*Hs*TIM. (**C**) Diagram of the Rg of the three complexes and apo-*Hs*TIM.

Molecular dynamics simulations were performed (Figure 4) to determine the stability of the complexes formed from the compounds **C6**, **E2** and **P9** at the *Hs*TIM interface. The RMSD values of apo-*Hs*TIM remained constant with a minimum fluctuation of 0.30 Å, a maximum of 2.49 Å, and a mean oscillation of 1.63 Å (Figure 4A). The RMSD value of the **C6**-*Hs*TIM complex ranged from 0.77 to 9.92 Å with a mean range of 7.58 Å. The RMSD value for the complex with **P9** was from 0.73 to 9.52 Å with a mean oscillation of 6.60 Å. The **E2**-*Hs*TIM complex presented a RMSD of 1.14 to 28.64 Å with a mean oscillation of 20.24 Å, with this being the most unstable compound. Figure 4B shows the RMSF plot with large fluctuations in most regions according to the RMSD pattern.

In addition, the Rg (Figure 4C) was determined for the apo-*Hs*TIM with values from 24.29 to 25.17 Å, with a mean oscillation of 24.68 Å. For the **C6**-*Hs*TIM and **P9**-*Hs*TIM complex, the Rg was very similar (24.35 and 24.36 Å) with a mean oscillation of 24.81 and 24.74 Å, respectively. **E2**-*Hs*TIM complex presented a minimum fluctuation of 24.32 Å and a maximum of 25.73 Å with a mean oscillation of 24.74 Å.

## 3. Materials and Methods

### 3.1. Control Compounds

Six compounds (**C1**–**C6**) with leishmanicidal or inhibitory activity against *Lm*TIM were sketched in ChemDraw and saved in SDF format. Subsequently, they were minimized and converted to pdbqt format with OpenBabel.

### 3.2. Molecular Docking Analysis

The crystallographic structure of the *Lm*TIM protein was obtained from the protein data bank (PDB) (http://www.pdb.org, accessed on 20 March 2020) [51] with the ID: 1AMK (resolution 1.83 Å), as well as the *Hs*TIM structure with the ID: 4POC. The structures were prepared for molecular docking with the UCSF Chimera 1.14.1 DockPrep tool (The Regents of the University of California, Oakland, California) [52]. Additionally, the prepare_receptor4.py script from MGLTools 1.5.6 (Center for Computational Structural Biology, La Jolla, California) was used to add AutoDock atom types and Gasteiger charges to them.

The prediction of potential binding sites on *Lm*TIM was first performed using the DoGSiteScorer tool (Center for Bioinformatics, Bundesstr, Germany) from the Proteins Plus server (https://proteins.plus/) (accessed on 23 March 2020) [53]. Subsequently, a blind molecular docking was performed. For this, the receptor was defined as rigid, and the docking protocol was setup up and performed with PyRx software, which works with AutoDock vina 1.1.2 [54]. For docking at the binding site, the conformational search space was determined by setting the coordinates to the center of the residues at the interface (X = −5.933, Y = −8.890, and Z = 7.297) using the PyRx software. The binding site on *Hs*TIM was determined by overlap between *Lm*TIM (ID: 1AMK) and *Hs*TIM apoprotein (ID: 4POC) using UCSF Chimera. Based on binding site prediction, the dimer interface residues were selected for a guided molecular docking on both *Lm*TIM and *Hs*TIM as previously described [55].

### 3.3. Ligand-Based Virtual Screening

LBVS was carried out by substructure search using the benzimidazole scaffold in the ZINC15 database (https://zinc15.docking.org/) (accessed on 28 March 2020) [56]. Subsequently, the structures were obtained using SMILES representations. Lipinski’s rule was applied using the OpenBabel program. Finally, the structures were prepared for molecular docking at the *Lm*TIM interface using the PyRx program, using the affinity energy of the compound **C6** (−9.2 Kcal/mol) as the cutoff. Subsequently, through the PLIP web server, an interaction profile was generated for each of the complexes [57]. With the scikit-learn library and the DataWarrior program (https://openmolecules.org/datawarrior/) (accessed on 23 March 2020) [58,59], the compounds were clustered according to their interaction profiles and the kind structure similarity. Finally, two compounds were selected based on cost and availability for evaluation against *L. mexicana* promastigotes and molecular dynamics analysis.

### 3.4. In Vitro Leishmanicidal Activity

The leishmanicidal assay was carried out following the procedure reported by Muñoz et al. [60] and Inchausti et al. [61]. The strain of L. mexicana MHOM/MX/2011/Lacandona, kindly donated by Dr. Ingeborg Becker Fauser of the UNAM, was used, which was maintained in Schneider culture medium with 10% fetal bovine serum (FBS); parasites in the log phase of their growth cycle were transferred to a microplate (96 wells; 1 × 10^6^ parasites/well). Stock solutions of DMSO (blank), pentamidine (positive control), and benzimidazole derivatives were diluted in Schneider’s medium to <100 μg/mL, added to the plate, and incubated at 27 °C for 72 h. IC_50_ values (µg/mL) were obtained using the Biostat 2009 statistical program. Subsequently, the results were converted to micromolar. The benzimidazole derivatives (**P9** and **E2**) were purchased from MolPort and worked up without further purification. The assay was performed in triplicate.

### 3.5. Molecular Dynamics Analysis

For the analysis of the molecular dynamics of compounds **P9** and **E2**, the open source software package GROMACS 5.1.2 [62] was used. Protein ID: 1AMK was parameterized in the AMBER03 force field (ff94/ff99 modification by Duan et al. [63] with the GROMACS pdb2gmx software package. The protonation state of the protein, pH 7, was previously calculated with the PROPKA tool implemented in UCSF Chimera. On the other hand, the topology of the compounds was generated with ACPYPE Server (http://webapps.ccpn.ac.uk/acpype accessed 18 May 2020) [64], which is based on the General Amber Force Field (GAFF). The system was a dodecahedron with periodic boundary conditions. In addition to containing the protein–ligand complex, it was filled with TIP3P water molecules and the number of ions (Cl^−^ or Na^+^) necessary to have a neutral charge in the system. Before running the dynamics, the system was energetically minimized using the steepest descent algorithm. Then, two equilibrium steps were performed with 1000 kJ/mol nm^2^ constraints on the movement of light and heavy proteins and atoms. The first stage was at constant pressure, implementing the frog-jump method and the v-rescale thermostat to bring the system from 0 to 300 K. The second stage was performed at constant temperature again with the frog-jump method, but now with the Berendsen barostat method to bring the system from 1 to 2 bar. Both stages achieved a duration of 100 ps. Once the system was balanced, molecular dynamics was performed with a trajectory of 100 ns for LmTIM and 120 ns for HsTIM, where interactions and long-range forces were calculated with the particle-mesh Ewald (PME) method, establishing the Lennard-Jones and Coulomb contributions at 1.2. nm, balancing the system with samples at 100 ps. The stability of the complexes was determined using the GROMACS software tools and the root mean square deviation (RMSD) between the α carbons and the ligand was obtained, and the root mean square fluctuation (RMSF) of α carbons, together with two-dimensional structure and the radius of gyration (Rg), were calculated.3.6. Analysis of Molecular Physicochemical Properties

Compounds **P9** and **E2** were analyzed in silico with the SwissADME website (http://www.swissadme.ch/, accessed 20 August 2021) [65] to determine their physicochemical and pharmacokinetic properties, as well as their hepatotoxicity from the ProTox-II server (https://tox-new.charite.de/protox_II/, accessed 24 October 2021) [66].

## 4. Conclusions

In this work, a virtual screening based on the benzimidazole scaffold, and a molecular docking directed at the *Lm*TIM dimer interface, allowed the prediction of 175 new benzimidazole derivatives with docking scores between 10.8 and −9.0 Kcal/mol on *Lm*TIM. The in vitro evaluation against the promastigote forms of *L. mexicana* determined that compound **E2** has better leishmanicidal activity than **P9** and a value similar to pentamidine. Finally, a low affinity (−5.8 Kcal/mol) for **E2** and the molecular dynamics studies at the *Hs*TIM interface suggest selectivity on *Lm*TIM. These results encourage us to continue the study of benzimidazole derivatives to obtain new and more selective leishmanicidal agents.

## Figures and Tables

**Table 1:** Docking scores and molecular interactions of compounds used as control ligands at the *Lm*TIM interface.

Chemical Structure of Control Ligands	
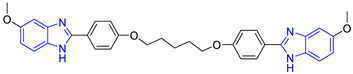 **C1** DS= −7.2 Kcal/mol ^1^ IC_50_ = 0.71 μM [24] HI: Ile69B, Ala70B, Lys71B, Glu105B, Ile109B; HB: Gln112B, Ala70B, Lys71B, Lys113B; π-S: Phe75B, Tyr102A, Tyr103A	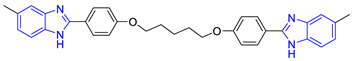 **C2** DS= −7.8 Kcal/mol ^1^ IC_50_ = 0.36 μM [24] HI: Ile69B, Ala70B, Lys71B, Glu105B, Ile109B; HB: Gln112B, Ala70B, Lys71B, Lys113B; π-S: Phe75B, Tyr103A
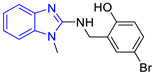 **C3** DS= −7.3 Kcal/mol ^1^ IC_50_ = 2.62 μM, ^2^ IC_50_ = 0.28 μM [25] HI: Ile69B, Ala70B, Lys71B, Glu105B, Ile109B, Phe75B; HB: Gln112B, Lys113B; π-S: Tyr103(A)	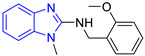 **C4** DS= −7.1 Kcal/mol ^1^ IC_50_ = 3.21 μM, ^2^ IC_50_ = 0.26 μM [25] HI: Ile69B, Lys71B, Glu105B, Ile109B; HB: Tyr103A π-S: Phe75B, Tyr103A
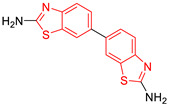 **C5** DS= −7.6 Kcal/mol 70% enzyme inhibition at 100 µM [10] HI: Glu105B, Ile109B; HB: Tyr103A	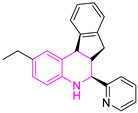 **C6** DS= −9.2 Kcal/mol ^1^ IC_50_ = 1.02 μg/mL, Cell line J774 = 187.0 μg/mL; SI = 183.3 [26] HI: Ile69B, Phe75B, Ala70B, Tyr102A, Tyr103A, Ile109B, Glu105B

^1^ Promastigotes, ^2^ Amastigotes, DS: Docking score, HI: Hydrophobic interactions, HB: Hydrogen bonds, π–S: π-stacking interactions, SI: Selectivity index.

**Table 2:** Lead compounds from each group obtained by SBVS grouped by interaction profile.

Group	Lead Compound	Group	Lead Compound
1 (17 compounds)	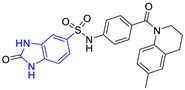 ZINC000030028937 (**P1**) DS= −9.6 Kcal/mol HI: Ala70B, Ile109B, Tyr102A, Ile69B, Lys71B; HB: Tyr103A, Lys71B, Ser72B, Ala74B; π-S: Phe75B, Tyr103A	6 (11 compounds)	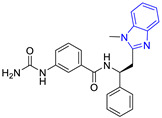 ZINC000010116378 (**P6**) DS= −9.3 Kcal/mol HI: Ile109B, Glu116B, Ala70B, Gln112B, Ile69B, Tyr102A, Phe75B; HB: Lys113B, Gln112B, Glu105B, Arg99B, Asn64B, Glu78B; π-S: Tyr103A, Tyr102A
2 (24 compounds)	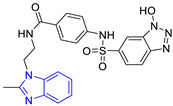 ZINC000010125646 (**P2**) DS= −9.6 Kcal/mol HI: Tyr103A, Tyr102A, Glu116B, Gln112B, Ile69B, HB: Ala70B, Lys113B; HB: Lys113B, Glu105B, Arg99B, Asn67B, Ala70B; π-S: Tyr103A, Tyr102A	7 (22 compounds)	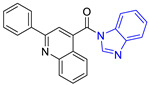 ZINC000000182545 (**P7**) DS= −9.4 Kcal/mol HI: Tyr102A, Ile69B, Phe75B, Pys71B, Ala70B, Ile109B, Glu105B; HB: Tyr103A; π-S: Tyr103A
3 (17 compounds)	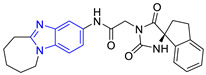 ZINC000030011443 (**P3**) DS= −9.6 Kcal/mol HI: Lys71B, Ile109B, Glu105B, Ile69B, Ala70B; HB: Lys71B, Gln112B, Ala70B; π-S: Tyr103A	8 (9 compounds)	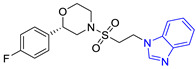 ZINC000030009197 (**P8**) DS= −9.8 Kcal/mol HI: Ile109B, Glu105B, Ile69B, Phe75B; HB: Tyr103A, Gln112B, Tyr102A; π-S: Tyr103A
4 (14 compounds)	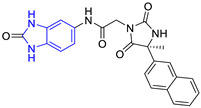 ZINC000010109617 (**P4**) DS= −10.3 Kcal/mol HI: Tyr103A, Ile109B, Phe75B, Ala70B, Glu116B, Gln112B, Lys71B, Ile69B; HB: Ala70B, Gln112B, Glu105B, Arg99B, Tyr103B, Lys113B	9 (23 compounds)	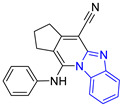 ZINC000000183176 (**P9**) DS= −9.4 Kcal/mol HI: Lys71B, Gln112B, Glu116B, Ala70B, Lys113B, Tyr103A, Tyr102A, Phe75B; HB: Gln112B, Lys113B; π-S: Phe75B
5 (19 compounds)	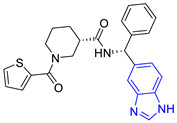 ZINC000040058994 (**P5**) DS= −9.4 Kcal/mol HI: Ile109B, Ala70B, Gln112B, Phe75B, Ile69B, Tyr102A, Tyr103A; HB: Lys113B, Tyr103B, Glu105B, Arg99B, Ala70B; π-S: Tyr103A	10 (19 compounds)	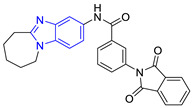 ZINC000030011418 (**P10**) DS= −9.7 Kcal/mol HI: Lys113B, Ala70B, Phe75B, Tyr102A, Ile69B, Ile109B, Tyr103A; HB: Lys113B, Ala70B, Lys71B, Gln112B, Glu105B; π-S: Tyr102A

DS: Docking score, HI: Hydrophobic interactions, HB: Hydrogen bonds, π–S: π-stacking interactions.

**Table 3:** Lead compounds from each group obtained by SBVS grouped by structure.

Group	Lead Compound	Group	Lead Compound
1 (20 compounds)	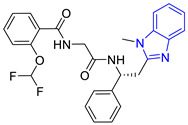 ZINC000010116253 (**E1**) DS= −10.8 Kcal/mol HI: Ile69B, Ile109B, Lys71B, Phe75B, Tyr102A; HB: Gln112B; π-S: Tyr103A	6 (4 compounds)	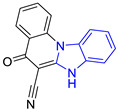 ZINC000000086631 (**E6**) DS= −9.0 Kcal/mol HI: Ile69B, Ile109B, Phe75B, Glu105B, Tyr102A; HB: Tyr102A; π-S: Tyr103A
2 (26 compounds)	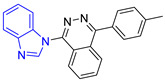 ZINC000000087857 (**E2**) DS= −9.4 Kcal/mol HI: Ile69B, Ile109B, Phe75B, Tyr103A, Gln112B, Glu116B; HB: Gln112B	7 (1 compound)	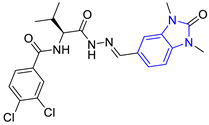 ZINC000010000495 (**E7**) DS= −9.7 Kcal/mol HI: Ile69B, Ala70B, Ile109B, Phe75B, Tyr102A, Tyr103A, Gln112B; HB: Ala70B, Tyr103A, Lys113B, Lys71B
3 (102 compounds)	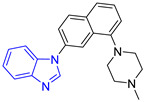 ZINC000000018961 (**E3**) DS= −9.3 Kcal/mol HI: Ile69B, Ala70B, Ile109B; HB: Ala70B, Gln112B; π-S: Phe75B, Tyr103A	8 (4 compounds)	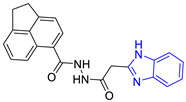 ZINC000010126510 (**E8**) DS= −9.5 Kcal/mol HI: Ile69B, Ile109B, Phe75B, Tyr102A, Tyr103A; HB: Gln112B, Tyr103A, Tyr102A; π-S: Phe75B, Tyr102A
4 (4 compounds)	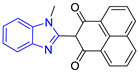 ZINC000000134117 (**E4**) DS= −9.3 Kcal/mol HI: Ala70B, Ile109B, Lys71B, Phe75B, Glu105B; HB: Gln112B, Lys113B; π-S: Tyr103A	9 (3 compounds)	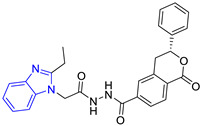 ZINC000010054868 (**E9**) DS= −9.4 Kcal/mol HI: Ile69B, Ile109B, Tyr103A; HB: Tyr102A; π-S: Phe75B; SB: Lys113B
5 (6 compounds)	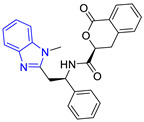 ZINC000010116541 (**E5**) DS= −9.2 Kcal/mol HI: Ile69B, Lys71B, Glu105B; HB: Gln112B; π-S: Phe75B, Tyr103A, Tyr102A	10 (2 compounds)	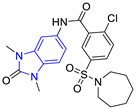 ZINC000020062149 (**E10**) DS= −9.1 Kcal/mol HI: Ile69B, Lys71B, Phe75B, Glu105B, Tyr103A; HB: Gln112B; π-S: Phe75B; Halogen: Lys71B

DS= Docking score, HI: Hydrophobic interactions, HB: Hydrogen bonds, π–S: π-stacking interactions, SB: Salt bridge.

**Table 4:** Leishmanicidal activity of benzimidazole derivatives against *L. mexicana* promastigotes.

Compound		Promastigotes IC_50_ (µM)
**P9**	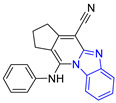	>148.63
**E2**	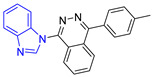	4.04 ± 0.39
Pentamidine		2.23 ± 0.79

**Table 5:** Calculated physicochemical and pharmacokinetic parameters of benzimidazole derivatives by SwissADME.

Physicochemical Properties	Compounds		Pharmacokinetic Properties	Compounds	
	P9	E2		P9	E2
MW (g/mol) < 500	324.38	336.39	Permeability BBB	Yes	Yes
Rotatable bonds < 10	2	2	GI Absorption	Elevated	Elevated
Hydrogen bond acceptors < 10	2	3	P-gp subtrate	Yes	Yes
Hydrogen bond donors < 5	1	0	CYP1A2 inhibitor	Yes	Yes
TPSA (Å^2^) < 140	53.12	43.60	CYP2C19 inhibitor	Yes	Yes
Log *P* < 5	3.97	4.31	CYP2C9 inhibitor	Yes	No
Log *S*	Moderately soluble	Moderately soluble	CYP2D6 inhibitor	Yes	No
			Hepatotoxicity	Inactive	Inactive

MW: molecular weight, TPSA: polar surface area, Log *P*: partition coefficient, Log *S*: solubility coefficient, Permeability BBB: permeability of the blood–brain barrier.

## Data Availability

Date is contained in the article.

## Supplementary Materials

The following supporting information can be downloaded at: https://www.mdpi.com/article/10.3390/ph16030390/s1, Figure S1: Molecular dynamics trajectory analysis of the E2-*Lm*TIM complex; Figure S2:Molecular dynamics trajectory analysis of the P9-*Lm*TIM complex; Figure S3: Interaction profile of the P9-*Lm*TIM complex during molecular dynamics analysis; Figure S4: Interaction profile of the E2-*Lm*TIM complex during molecular dynamics analysis.
