# Supplementary material for: Virtual Screening of Benzimidazole Derivatives as Potential Triose Phosphate Isomerase Inhibitors with Biological Activity against Leishmania mexicana
Source: Pharmaceuticals (Basel). 2023 Mar 3;16(3):390. doi: 10.3390/ph16030390 (PMC10058926; doi:10.3390/ph16030390)
Supplement: Supplementary file 1 [file pharmaceuticals-16-00390-s001.zip › pharmaceuticals-2215718-supplementary-fina.pdf]

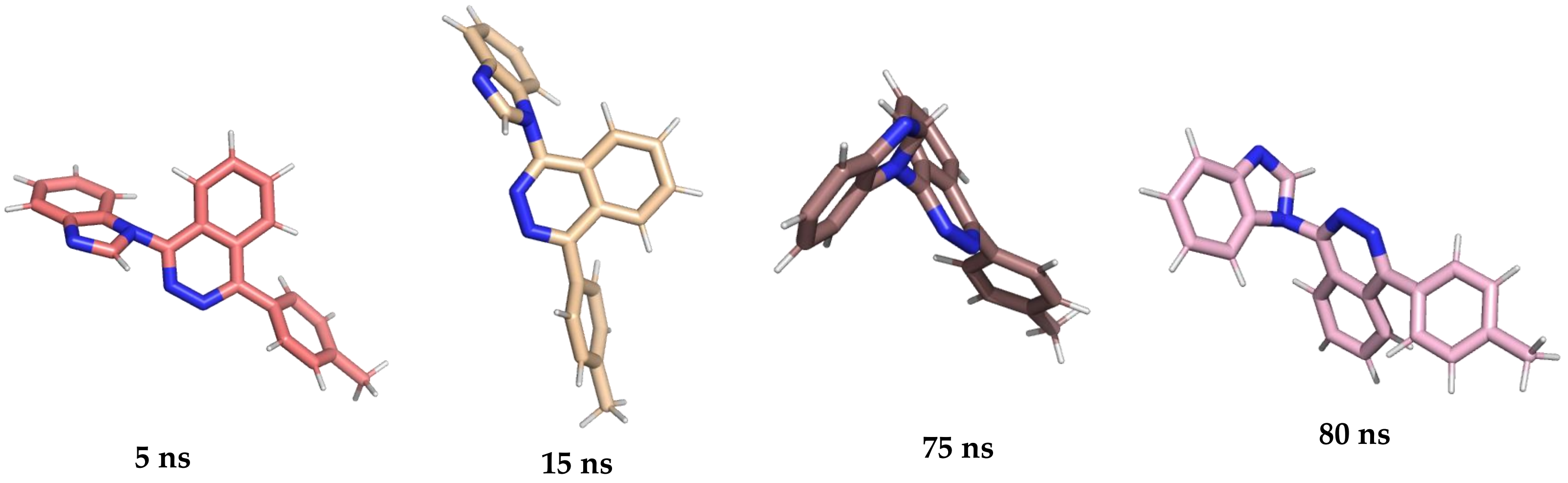

Figure S1. Molecular dynamics trajectory analysis of the E2-LmTIM complex.

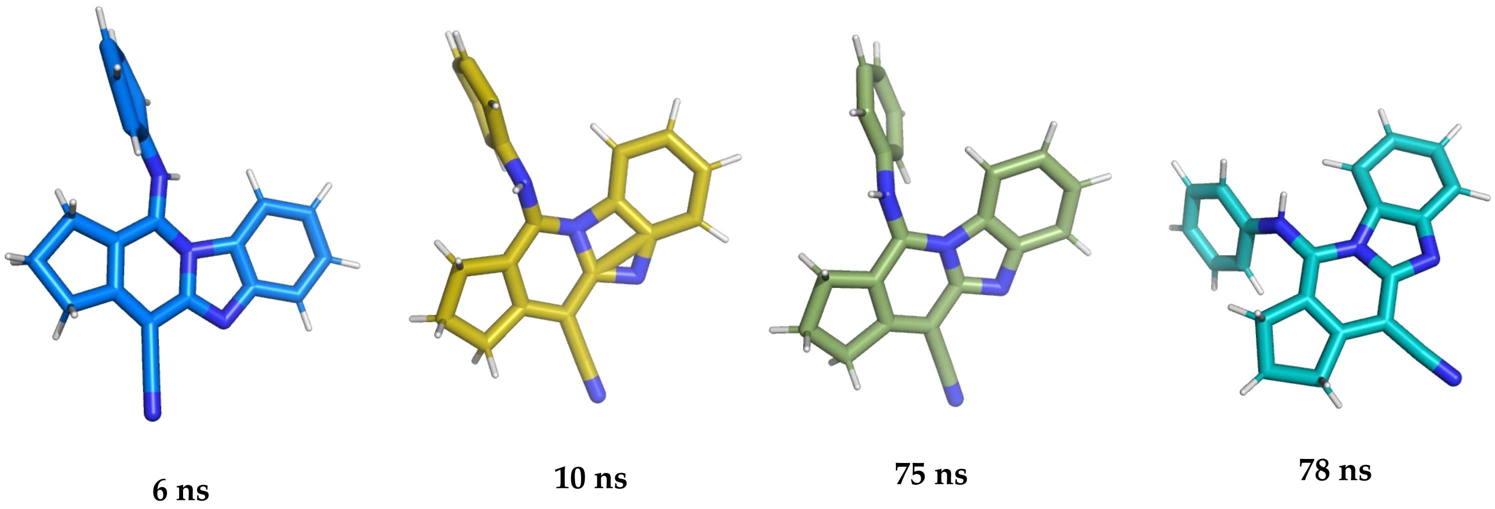

Figure S2. Molecular dynamics trajectory analysis of the P9-LmTIM complex.

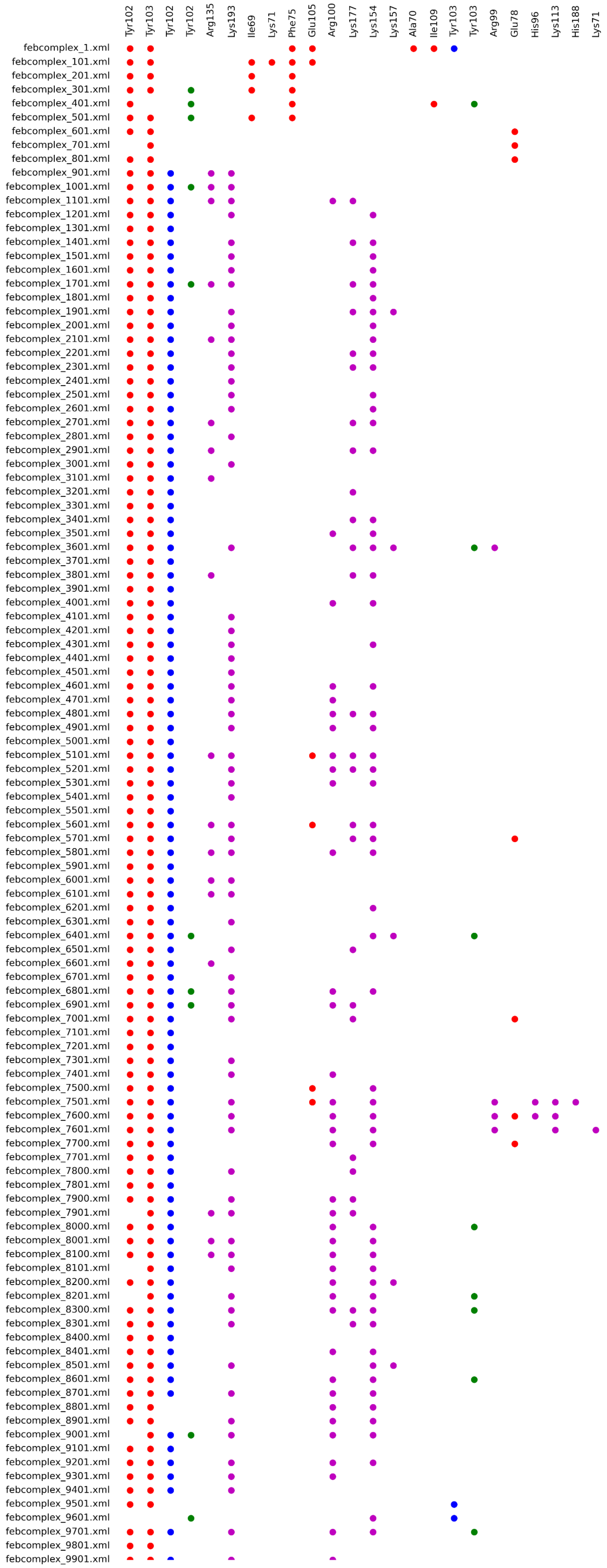

Figure S3. Interaction profile of the P9-*Lm*TIM complex during molecular dynamics analysis.

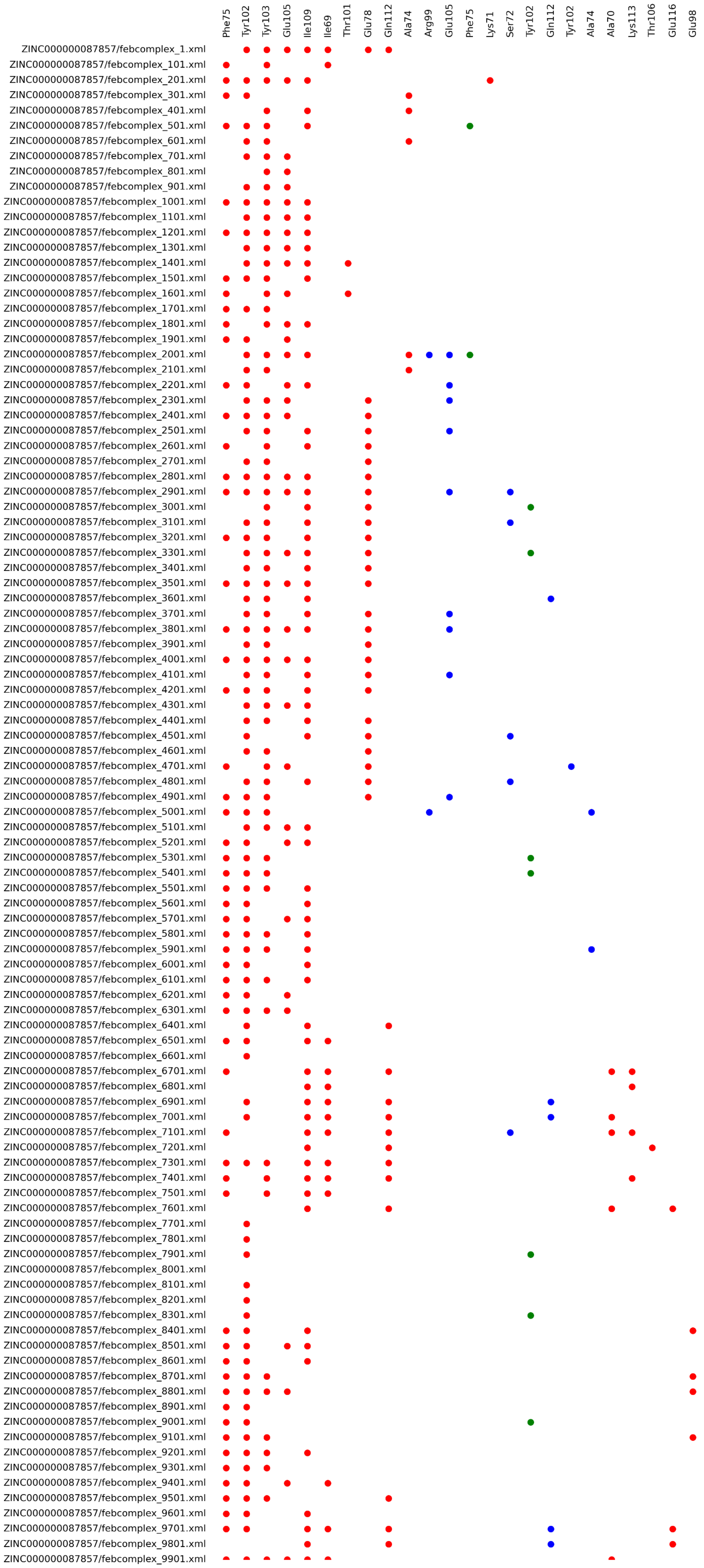

Figure S4. Interaction profile of the E2-*Lm*TIM complex during molecular dynamics analysis.
